# Developing an Analytical Model and Computing Tool for Optimizing Lapping Operations of Flat Objects Made of Alloyed Steels

**DOI:** 10.3390/ma13061343

**Published:** 2020-03-16

**Authors:** Tudor Deaconescu, Andrea Deaconescu

**Affiliations:** Department of Industrial Engineering and Management, Transilvania University of Brasov, 500036 Brasov, Romania; deacon@unitbv.ro

**Keywords:** lapping, spherical abrasive grain model, material removal rate, software tool

## Abstract

Lapping is a finishing process where loose abrasive grains contained in a slurry are pressed against a workpiece to reduce its surface roughness. To perform a lapping operation, the user needs to set the values of the respective lapping conditions (e.g., pressure, depth of cut, the rotational speed of the pressing lap plate, and alike) based on some material properties of the workpiece, abrasive grains, and slurry, as well as on the desired surface roughness. Therefore, a mathematical model is needed that establishes the relationships among the abovementioned parameters. The mathematical model can be used to develop a lapping operation optimization system, as well. To this date, such a model and system are not available mainly because the relationships among lapping conditions, material properties of abrasive grains and slurry, and surface roughness are difficult to establish. This study solves this problem. It presents a mathematical model establishing the required relationships. It also presents a system developed based on the mathematical model. In addition, the efficacy of the system is also shown using a case study. This study thus helps systematize lapping operations in regard to real-world applications.

## 1. Introduction

Surface lapping has been known for thousands of years as a procedure used for the manual finishing of stone objects [1]. For a long period of time, lapping remained a manual process and was considered an art, a craft rather than a science, due to the stochastic, often unpredictable nature of the results [2]. Generally, surfaces were lapped without applying certain rules, merely by combining work parameters based on the operator’s experience. At present manual lapping is rare and has been replaced by mechanized and automated processes.

There are some components (mechanical seals, components of pacemakers, measuring instruments, and surgical devices, cutting tools, hard disk drive heads, guiding surfaces of machine tools, and pistons of pumps) which are frequently used in automotive, aircraft, manufacturing, computer, and electronic industries. In most cases, the performances of these components depend on both form accuracy and surface finish. Therefore, if the form accuracy and surface finish of the abovementioned components do not meet the desired level after machining (e.g., turning, grinding, and alike), lapping (and in some cases followed by polishing) is performed. Therefore, how the form accuracy and surface finish of a workpiece change due to lapping is an important issue of research and implementation.

In lapping, loose abrasive grains mixed with slurry are pressed against a workpiece to reduce its surface roughness as well as its form accuracy (e.g., flatness). To perform a lapping operation, the user needs to set the values of the respective lapping conditions (e.g., pressure, depth of cut, the rotational speed of the pressing lap plate, and alike) based on some material properties of the workpiece, abrasive grains, and slurry, as well as on the desired surface roughness. Therefore, a mathematical model is needed that establishes the relationships among the abovementioned parameters.

A relatively reduced amount of specialist study has been dedicated to surface lapping. The literature features no general computational relationships linking the various parameters that impact the outcome of lapping. The values of the output quantities like roughness, superficial layer hardness, or material removal rate are presented strictly for concrete cases, and the results cannot be generalized. At present, lapping is a process that lacks digital support, including a database of practice-based information that would enable machining process planning and optimization.

Within this context, the paper addresses this gap in knowledge by putting forward an algorithm for the modeling of the lapping process of flat surfaces and a software tool developed as a support for specialists. The main objective of the paper is to make available to industrial specialists concerned with lapping technologies, a working instrument that simplifies and significantly reduces the duration for calculating output quantities that are the optimized machining parameters for flat lapping. Chapter 2 presents the principle of processing and data on the state-of-art in this field. The next chapter discusses the computational methodology of the main output quantities of a lapping system based on the spherical model considered for the abrasive grains. Further, in this chapter, a software application is described developed for the planning and optimization of the process by means that, the operator can set the optimum work parameters swiftly. The paper ends with a chapter of conclusions.

## 2. Principle of Lapping and Related Works 

In lapping, the machining allowance is small (< 50 μm), hence the similarity of such machining to the phenomenon of wear by abrasion [3]. It is not abrasion only that removes the surplus material layer, but to a smaller extent, also the corrosion caused by the working medium and the occurrence of fatigue [4]. The coexistence of all these processes needs detailed study and a thorough understanding of the numerous factors that contribute to the removal of material.

**Figure 1:**
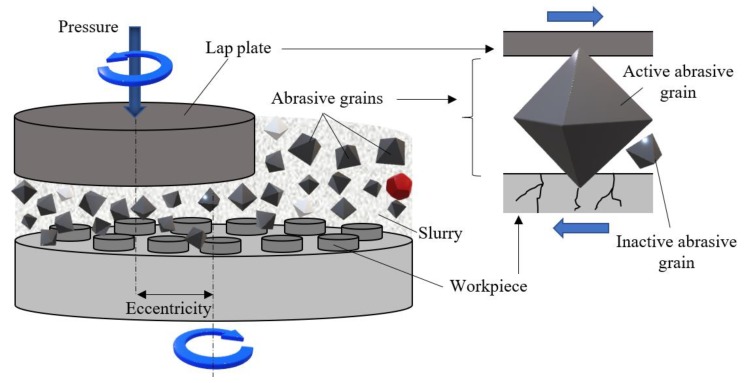
Principle of lapping.

The lapping system can be assimilated to a tribosystem consisting of the elements shown in Figure 1:

The tribosystem consists of the lap plate, one or more workpieces that are machined simultaneously, and the semi-liquid medium known as slurry. Each of these elements cumulates a number of characteristics like hardness, surface roughness, type of material, state of the surface layer, graining, etc. All these characteristics are completed by parameters pertaining to the kinematics of the machine-tool (machining speed, lap plate pressure, eccentricity between the lap plate axis and the axis of rotation of the workpiece support, etc.).

Lapping entails the use of a semi-liquid working medium (lapping paste or slurry) consisting of a carrier fluid that holds the abrasive grains in free distribution. The lapping system does not use a tool in the classical sense. Cutting is achieved by the cumulated action of a large number of grains held by a carrier fluid, hence tool edges being geometrically undetermined. As the slurry is fed continuously into the gap between the lap plate and the workpiece, there are always unused (inactive) grains in the work area. At the same time, the slurry also provides cooling and ensures the removal of the chips resulted from machining.

Material removal is achieved by the larger grains, known as active grains, the tips of which penetrate into the workpiece surface. [3]. Due to the continuous relative movement of lap plate and workpieces, the free-rolling grains are active and inactive in turn. The penetration depth of each grain depends on the pressure force exerted upon it by the lap plate. Microscopic cracks occur at the contact of grains and workpiece surfaces that, in time, merge and generate micro-craters of a volume that represents 2 to 5% of that of an abrasive grain [5,6].

To date, published research on lapping discusses only specific cases like the machining of ceramic materials, optical glass, SiC wafers, sapphire layers, etc. Thus, for example, paper [7] determines the machinability of several ceramic materials for different loads, working speed, and utilized abrasive materials. The experimental results showed that different slurry compositions hold relevance only for small lap plate pressures, while for greater pressures, the impact on the material removal rate is insignificant. Paper [8] presents the results of oxide ceramics lapping, with a focus on the influence of the contact pressure. Doubling the pressure from 25 to 50 kPa yields an increase of the removed material rate, a decrease of abrasive material consumption, and improved machined surface roughness.

In paper [9], the authors analyze the material removal rate in the lapping process of glass and reach the rather obvious conclusion that compared to sharp grains, used abrasive grains remove a smaller quantity of surplus material, while the duration of machining also impacts the volume of removed material.

Research concerning the finishing of SiC wafers presented in [10] proposes a four-step lapping process consisting of four stages of both-side lapping with different grit-size abrasives. The application of the proposed procedure yielded an outstanding quality of the machined surfaces, with roughness values (arithmetic mean deviation of the profile) reaching *Ra* = 0.168 nm. Other significant results related to the machining of SiC wafers are given in [11].

The double-sided lapping behavior of sapphire substrates using fixed diamond abrasive pads is evaluated in [12,13]. The conclusion shows that the removal rate is highly sensitive to lap plate pressure and abrasive grain size.

The kinematics of lapping machines has also been the object of research. Paper [14] proposes a system of simultaneous two-sided lapping in the planetary motion of the cylindrical surfaces of bearing rollers. The conclusion showed that the rolling speed of rollers only depends on the rotation speed of the lower plate rather than on that of the upper plate. Paper [15] contributes to the study of the trajectories of the abrasive grains between the lapping plate and the workpiece. It is shown that the lapping trajectory can be superposed periodically when a rotation ratio is a rational number.

Paper [16] studies the influence of various abrasive materials on the performance of lapping stainless steel and bronze and arrives at the conclusion that material removal rates were not influenced significantly by using different abrasives. Further, it was observed that surface roughness and flatness improve with the hardness of the abrasive. 

Regarding the material removal rate (*MRR*) in lapping many researchers rely on the abrasion/wear-based Preston’s equation (*MRR = K∙p∙vr*), where *K* is Preston’s coefficient, and the predominant input quantities are lap plate pressure (*p*) exerted on the workpiece and the relative speed (*vr*) of the two components [17]. Experimental research conducted by other researchers, however, proved that also important for calculating *MRR* are the characteristics of the abrasives or the lap plate material and its surface quality (roughness). It was further shown that the dependence of *MRR* on the various input quantities is not linear and that Preston’s equation does not explain such behavior.

The above considerations reveal the lack of a unitary approach to lapping, expressed by general computational relationships for the various work parameters. For this reason, this paper puts forward a new method for determining the technical time norm, the cutting parameters, and the material removal rate in flat lapping. This new method was materialized by conceiving a software tool that helps operators with the swift, automated planning of the machining process.

## 3. Modeling the Lapping of Flat Surfaces

The complexity of the phenomena occurring simultaneously during lapping requires the use of models for a true description of the machining process. Models help in identifying existing correlations between numerous parameters that influence machining and allow moving to the next step of automated design and optimization of the production process.

Literature includes several models of abrasive erosion focused on grain shape. Best known shapes are conical, spherical, diamond, prismatic, the Cassini oval, etc. [18,19,20]. The shape of abrasive grain is extremely complex and cannot be reduced to one of the above models as each grain is different from any other grain, the abrasive process has to be analyzed based on a model that reproduces reality as closely as possible.

In [1] and [3], lapping is described as a very low-speed process where the abrasive grains will first roll, slide, and then the actual chipping action starts. First, the abrasive grains roll along the workpiece, and then slide and with the application of load, the pressure on the grains constantly rises, leading to a chipping action (dislodging of miniature chips from the material). 

According to these descriptions, lapping is a process characterized by static loads that are applied progressively so that inertial forces are negligible. There are no dynamic loads involved that are applied with a certain velocity or abruptly, as is the case, e.g., in an abrasive jet or ultrasonic abrasive machining.

Within this context, the authors have formulated hypotheses on the basis of that the static model of grain penetration into the workpiece was conceived. These hypotheses include:

- The large active abrasive grains roll slowly between the lap plate and the workpiece;
- In turn, the tip of each grain penetrates the workpiece surface, what is a slow and shock-free process;
- The pressure is applied to the abrasive grains progressively, in a shock-free manner.

**Figure 2:**
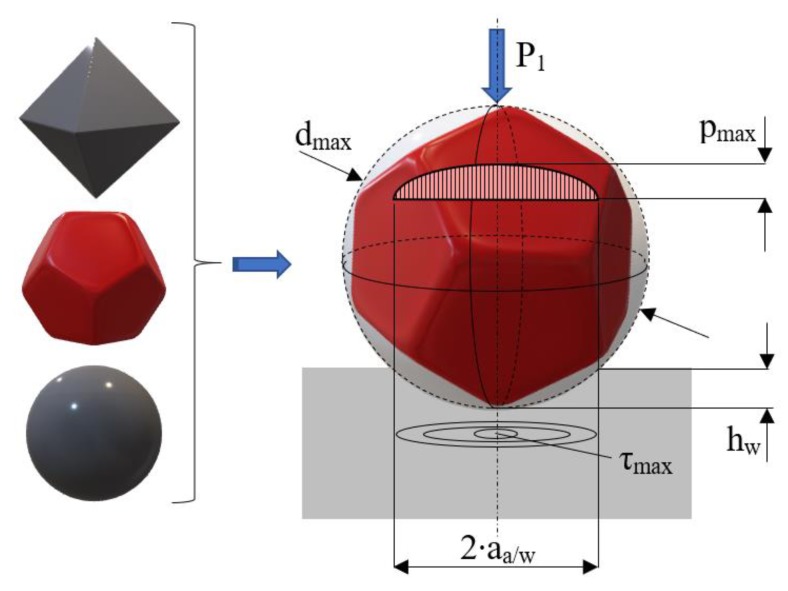
Spherical model of the abrasive grain.

In lapping abrasive grains are required to have an isometric (compact) shape in order to avoid their orientation by preferential directions. Based on this requirement, the proposed mathematical model uses the following hypotheses [21]:

- The abrasive grains are spherical (Figure 2). A spherical shape was adopted starting from research conducted by Moon, who proved by means of a scanning electron microscope (SEM) that the shape of the abrasive grains used in lapping is approximately round [22]. On their turn, Luo and Dornfeld proved that in calculating *MRR* the spherical model of abrasive grains yields the results closest to reality [23].
- According to the theory of elasticity, the Hertzian contact stress formulae apply to the contact area between the abrasive grain and the processed surface;
- The materials of the workpiece and the lap plate are isotropic, and the contact stress is uniformly distributed;
- The phenomenon of cold-hardening is neglected;
- The quantity of settled material around the grain is neglectable.

Figure 2 also illustrates the distribution of stress in the contact area between abrasive grain and the processed surface. The maximum shear stress of the machined material, *τ_max_*, is calculated by Equation (1) [24]:

(1) $$\tau_{max}=0.47\cdot p_{med}=0.47\cdot \frac{P_{1}}{\pi \cdot a_{a/w}^{2}}$$

where *p_med_* is the average contact pressure, and *a_a/w_* is the radius of the contact circle between the abrasive grain and the machined material.

The theoretical shear strength of a material is calculated by Equation (2) [24]:

(2) $$\tau_{t}=\frac{G_{w}}{k}$$

where *G_w_* is the transverse elastic modulus of the machined material, and *k* is a coefficient that can have the following values [24]:

- *k* = 2∙π, for metals (Al, Cu, Fe);
- *k* = 0.8∙π, for Si, Ge, diamond.

When the maximum shear stress exceeds the theoretical shear strength *τ_t_*, a tangential sliding of the material is initiated that causes a crack in it. By multiplying the actions of the abrasive grains, the number of cracks increases, the cracks intersect, and consequently, the material removal process is developed.

The equations above indicate that in metals the tangential sliding of the material occurs when:

(3) $$\tau_{max}=0.47\cdot \frac{P_{1}}{\pi \cdot a_{a/w}^{2}}>\frac{G_{w}}{2\pi }$$

wherefrom the value of force *P_1_* can be calculated that has to be applied to an abrasive grain so that tangential sliding occurs (*G_w_* = 81 GPa for steel):

(4) $$P_{1}>86.17\cdot a_{a/w}^{2}$$

The radius of the contact circle between the abrasive grain and the machined metal *a_a/w_* is calculated by Equation (5) [25]:

(5) $$a_{a/w}=\sqrt[{3}]{\frac{3\cdot P_{1}\cdot d_{max}\cdot K_{a/w}}{4}}$$

where *d_max_* is the maximum diameter of the abrasive grains, and *K_a/w_* is a constant depending on the materials of the grain and of the lapped workpiece:

(6) $$K_{a/w}=\frac{1}{2}\cdot \left(\frac{1-\nu_{a}^{2}}{E_{a}}+\frac{1-\nu_{w}^{2}}{E_{lp}}\right)$$

where *ν* are the Poisson’s ratios, and *E* are the longitudinal elastic moduli (Young’s moduli). The indices *a* and *w* of the two quantities refer to the material of the abrasive grain and of the machined surface, respectively.

By introducing Equation (5) into (4), the value of force *P_1_* can be determined:

(7) $$P_{1}>86.17^{3}\cdot {\left(\frac{3\cdot d_{max}\cdot K_{a/w}}{4}\right)}^{2}$$

The radius *a_a/w_* of the contact circle can also be computed geometrically according to Figure 2 by Equation (8):

(8) $$a_{a/w}=\sqrt{d_{max}\cdot h_{w}-h_{w}^{2}}$$

From the above relationship follows a quadratic Equation (9) the solutions of which yield the penetration depth of an abrasive grain into a steel workpiece (*h_w_*):

(9) $$h_{w}^{2}-h_{w}\cdot d_{max}+a_{a/w}^{2}=0$$

The abrasive grain penetrates not only into the workpiece but also into the lap plate. The abrasive grain—lap plate contact is analyzed similarly to the above case, taking into account the material characteristics of this friction pair. Lap plates are typically made from cast iron that has a smaller Young’s modulus than steel. For this reason, in the case of cast iron, a larger value will result in the force *P_1_* to be applied to an abrasive grain in order to cause tangential sliding. As the useful effect of lapping is measured on the workpiece and not on the lap plate, the value of the downward force (4) necessary for machining the workpiece will be used for the computations related to the abrasive grain-lap plate contact. In this case, the radius of the contact circle between abrasive grain and the lap plate is:

(10) $$a_{a/lp}=\sqrt[{3}]{\frac{3\cdot P_{1}\cdot d_{max}\cdot K_{a/lp}}{4}}$$

where *K_a/lp_* is:

(11) $$K_{a/lp}=\frac{1}{2}\cdot \left(\frac{1-\nu_{a}^{2}}{E_{a}}+\frac{1-\nu_{lp}^{2}}{E_{lp}}\right)$$

where the index *lp* refers to the material of the lap plate.

Similarly to the abrasive grain/workpiece contact, also in the case of the abrasive grain/lap disc contact the penetration depth of the abrasive grain into the lap plate *h_D_* can be calculated as the solutions of Equation (12):

(12) $$h_{lp}^{2}-h_{lp}\cdot d_{max}+a_{a/lp}^{2}=0$$

**Figure 3:**
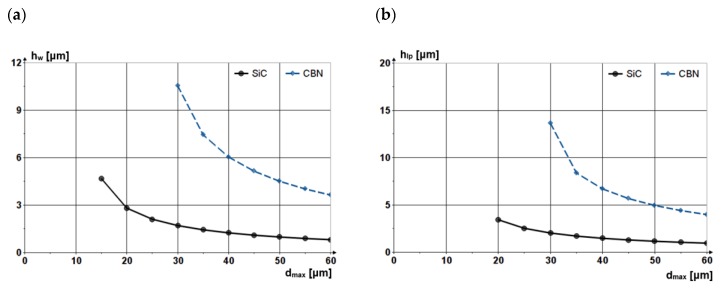
(**a**): Dependences of the penetration depths of the abrasive grains into the workpiece (*h_w_*) and (**b**): into the lap plate (*h_lp_*) versus grain diameter and grain material.

Figure 3 shows the penetration depth of the abrasive grain made from SiC (silicon carbide) and CBN (Cubic Boron Nitride), respectively, into a steel workpiece and a cast iron lap plate versus the dimensions of the abrasive grains:

It can be noticed that the difference in hardness of the two grain materials (*H_SiC_ < H_CBN_*) generates different penetration depths, as a grain made from CBN erodes more material.

**Figure 4:**
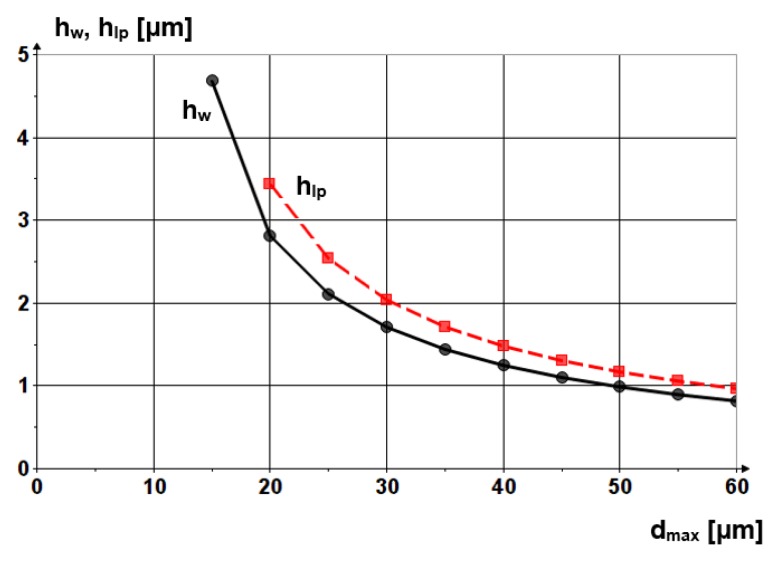
Penetration depths of abrasive grain made from SiC into the workpiece and the lap plate versus the grain diameter.

Figure 4 shows the penetration depths into the workpiece and into the lap plate of an abrasive grain made from SiC versus grain diameter. It can be noticed that the grain penetrates more into the lap plate because of the difference in Young’s moduli of the materials of the two components. Nevertheless, the abrasive grains do not erode the lap plate, grain penetration causing only elastic and plastic strain. No material chips are removed from the lap plate surface because the force exerted by the grain is not sufficient to cause tangential sliding of the material.

**Figure 5:**
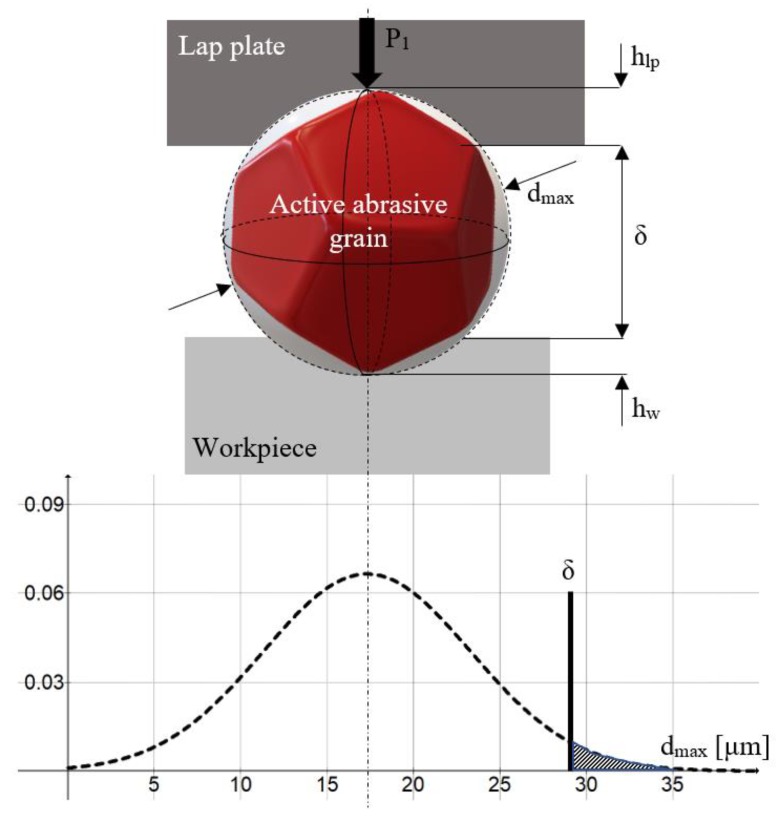
Distribution of F400 abrasive grain sizes.

Material is removed from the workpiece surface only by large grains, known as active grains, of sizes included in the interval [*δ; d_max_*] (Figure 5). The other, inactive grains of dimensions smaller than *δ*, float in the gap between lap plate and workpiece. The accurate prognosis of the number of abrasive grains involved in machining is important for the determination of *MRR*.

Figure 5 presents the working diagram of an active abrasive grain; the powder consists of F400 micro grains of an average grain size *d_med_* = 17.3 ± 1 µm. According to ISO 8486-2, the theoretical grain sizes in the 3% and 94/95% points of the grain size distribution curve (*d_s3_* and *d_s94/95_*) are of 32 and 8 µm, respectively [26]. Figure 5 illustrates this distribution.

The hashed area on the right-hand side of the graph indicates the fraction of abrasive grains that actually participate in the cutting process (grains of size > *δ*). This fraction is calculated by Equation (13):

(13) $$F_{p}=0.5-\frac{1}{\sqrt{2\cdot \pi }}\overset{z}{\underset{0}{\int }}e^{-\frac{z^{2}}{2}}\cdot dz$$

whereby the change of variable the so-called normed normal variable *z* is calculated by Equation (14):

(14) $$z=\frac{\delta -d_{med}}{\sigma }$$

For F400 abrasive material made from SiC, the calculated value of *F_p_* is 0.05448, meaning that merely 5.448% of the abrasive grains participate in the cutting process.

The volume of abrasive material necessary for lapping is calculated based on the formula of abrasive concentration in the lapping slurry:

(15) $$C=\frac{m_{a}}{m_{a}+m_{l}}=\frac{\rho_{a}\cdot V_{a}}{\rho_{a}\cdot V_{a}+\rho_{l}\cdot V_{l}}$$

where *m*, *ρ* and *V* are the mass, density, and the volume of the abrasive (index *a*) and of the carrier fluid (index *l*), respectively.

Denoting the area of the surface to be machined by *S* and the machining allowance by *h*, the volume, volume flow rate, and the mass of the abrasive to be fed to the working area over the entire duration of machining is calculated by Equations (16) to (18):

(16) $$V_{a}=\frac{\rho_{l}\cdot C\cdot S\cdot \left(h+\delta \right)}{\rho_{a}\cdot \left(1-C\right)+\rho_{l}\cdot C}\cdot \frac{1}{100\cdot F_{p}}$$

(17) $$Q_{a}=\frac{\rho_{l}\cdot C\cdot S\cdot \left(h+\delta \right)}{\rho_{a}\cdot \left(1-C\right)+\rho_{l}\cdot C}\cdot \frac{1}{100\cdot F_{p\cdot t}}$$

(18) $$m_{a}=\rho_{a}\cdot V_{a}$$

The material removal rate (*MRR*) is also determined in dependence on the magnitude of the machining allowance *h* and the duration of machining *t*:

(19) $$MRR=\frac{h}{t}$$

The proposed spherical model, together with all equations deriving from it, underlies the development of a technological software tool that enables swift automated planning of flat surface lapping.

## 4. Flat Lapping Technological Software Tool (FLaT)

The software tool developed for flat lapping (FLaT) includes specially conceived algorithms, dedicated databases, and a friendly user interface. Within a complete software application, FLaT is the component designed to provide the end-user with the necessary technical documentation. The output quantities provided by the software tool are the machining time (min), the height of the removed layer of material (μm), the material removal rate (μm/min), the flow rate of the abrasive (mm^3^/min) and the mass of the abrasive (g).

The processing time was calculated by Equation (20):

(20) $$t=\frac{\sqrt[{vR}]{Ra_{f}}-\sqrt[{vR}]{Ra_{i}}}{\sqrt[{vR}]{K_{R}\cdot e^{xR}\cdot C^{yR}\cdot vr^{zR}\cdot p^{uR}}}$$

with the following notations [4]:

- *Ra_i_*, *Ra_f_*—workpiece surface roughness (arithmetic mean deviation of the profile) before (*Ra_i_*) and after (*Ra_f_*) lapping, respectively;
- *K_R_, vR, xR, yR, zR, uR*—experimentally determined coefficients and exponents. The values of these coefficients and the characteristics of machined materials are included in a database that is part of the software tool (FLaT);
- *e*—eccentricity of the lap plate
- *C*—concentration of the abrasive;
- *vr*—relative machining speed;
- *p*—pressure exerted by the lap plate onto the workpiece.

The height of the removed material layer is calculated by a relationship of the form of Equation (21):

(21) $$h=K_{h}\cdot e^{xh}\cdot C^{yh}\cdot vr^{zh}\cdot p^{uh}\cdot t^{vh}$$

where *K_h_, vh, xh, yh, zh, uh* are experimentally determined coefficients and exponents. The values of these coefficients are included in the same database that comprises the machined materials [4].

**Figure 6:**
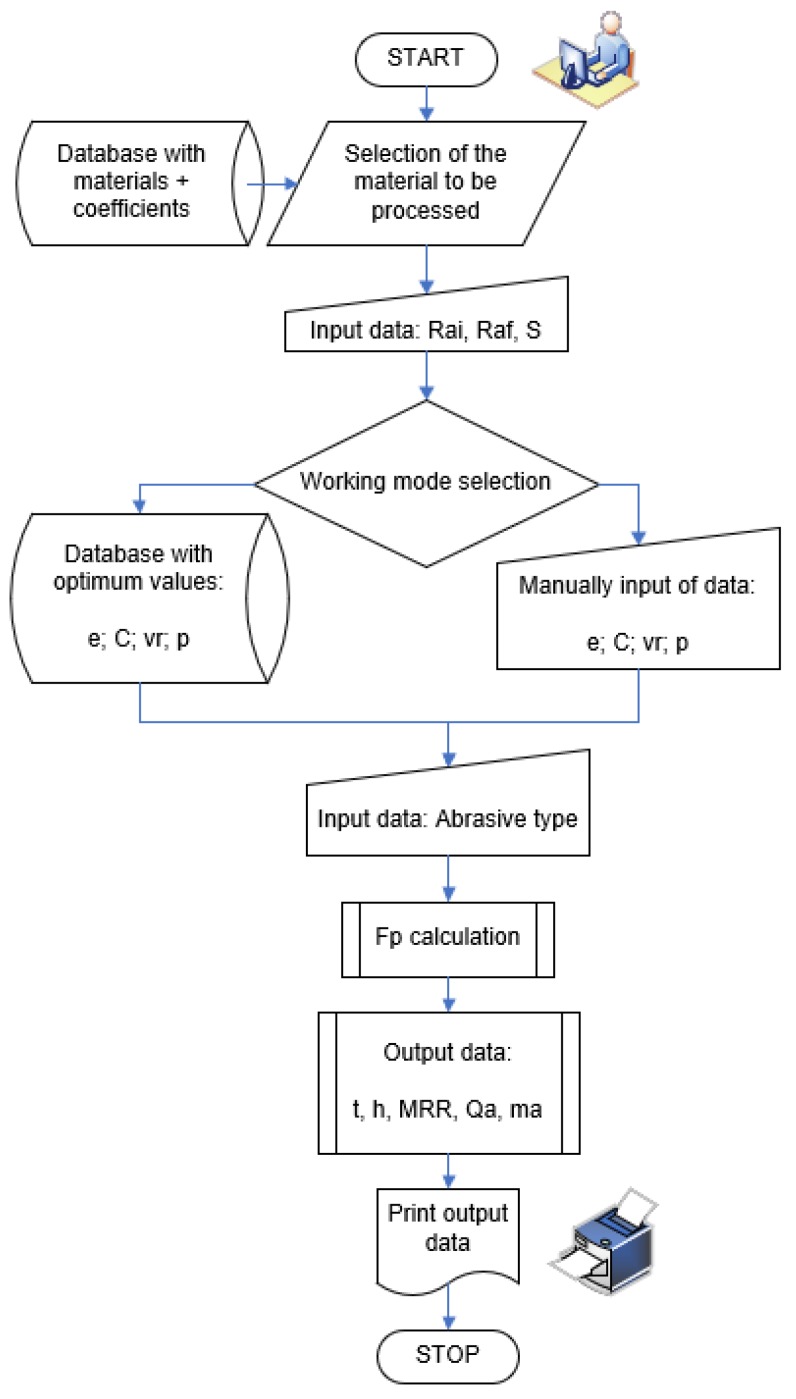
Flowchart of the software tool.

**Figure 7:**
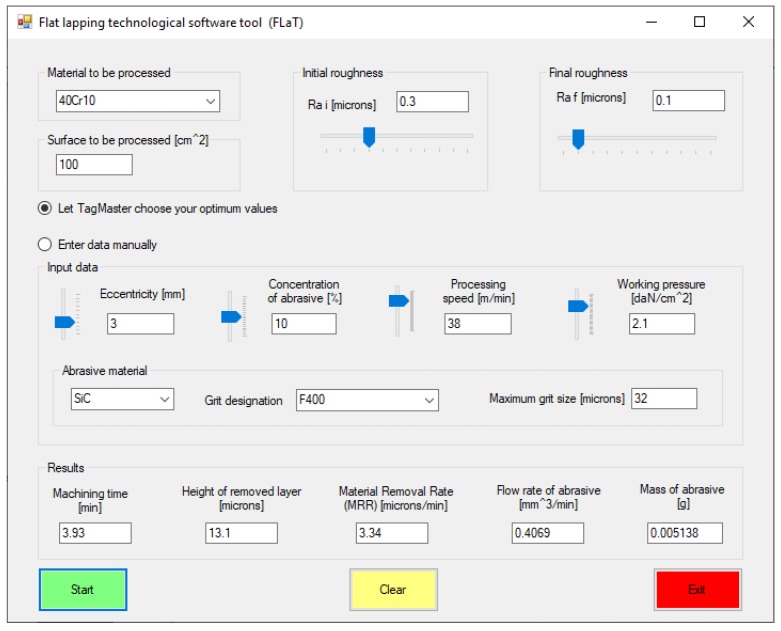
The user interface of the software tool.

Figure 6 shows the flowchart of the flat lapping process:

The software tool was developed as a Visual Basic.NET application and provides the graphic user interface shown in Figure 7:

The initial step requires the user to select the material to be machined. For this, the software tool makes available a database with information on several types of steel (including the coefficients and exponents from Equations (20) and (21)). The information is retrieved by connecting to an MS-Access file. 

After entering the values of the initial and final roughness and of the machined surface area, the user is required to select the working mode by means of radio buttons; thus, optimized input data can be used provided by a dedicated application known as TagMaster [27], or input data can be entered manually. By using the optimum values provided by TagMaster from its own built-in database, the machining process is rendered robust, which is insensitive to external noise factors.

The next step is the selection of the abrasive material and its respective graining. Upon pressing the Start button, the desired results are displayed.

A series of conclusions followed upon running the software tool:

- The duration of processing is greater when eccentricity and relative speed increase or for lower abrasive concentrations and working pressures;
- The machining allowance increases when increasing eccentricity and relative speed. The machining allowance is lesser impacted by modifications of working pressure and abrasive concentration;
- The material removal rate (*MRR*) increases significantly when working pressure and abrasive concentration are increased, and has insignificantly modified values for variations of the relative speed and eccentricity. The most significant increase of *MRR* is influenced by the downward pressure between the lap plate and the workpiece, while the relative speed has the smallest impact.
- The flow rate of the abrasive increases with increasing abrasive concentration and working pressure, or with decreasing relative speed and eccentricity.

**Figure 8:**
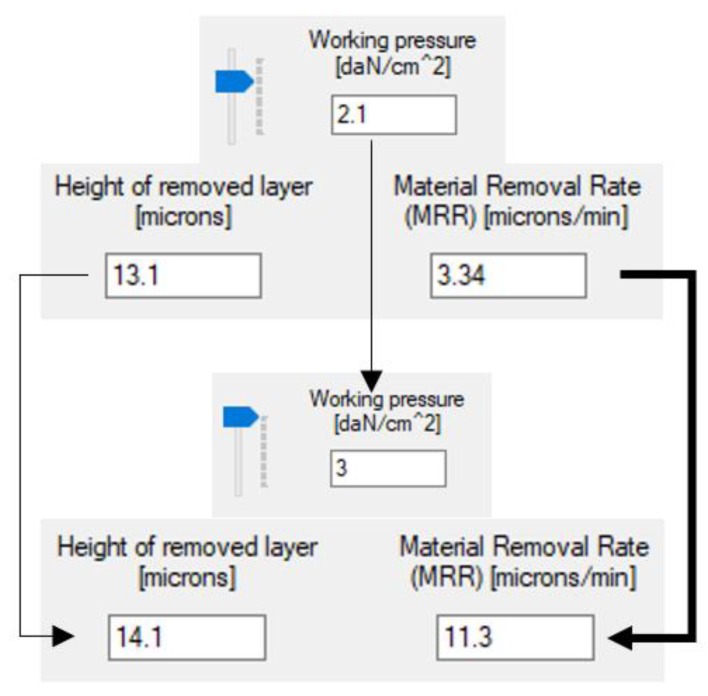
Variation of the machining allowance and material removal rate with the increase of working pressure.

**Figure 9:**
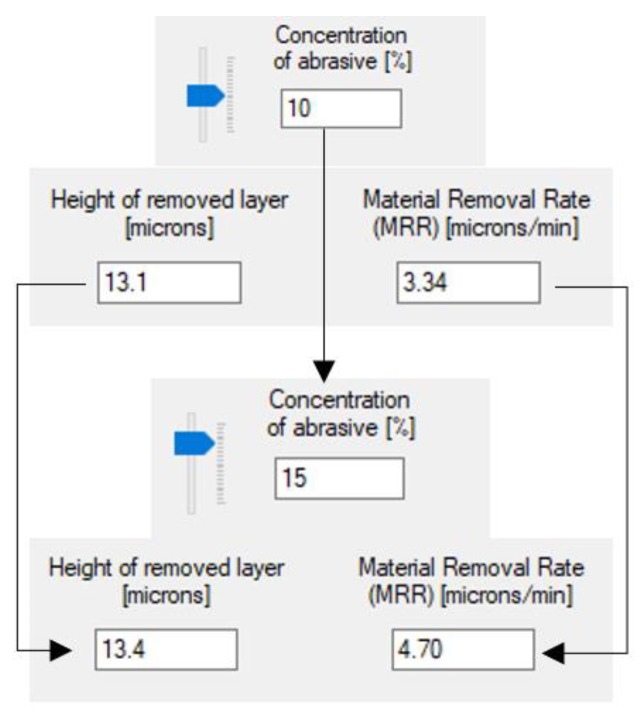
Variation of the machining allowance and material removal rate with the increase of abrasive concentration.

Figure 8 and Figure 9 illustrate the utilization of the software tool in a number of concrete cases, as well as the variation of the machining allowance and *MRR* in the dependence on working pressure and concentration.

It can be noticed a 50% increase in working pressure and abrasive concentration has an insignificant impact on the machining allowance. *MRR*, however, increases more than three times with growing pressure and by about 40% for higher concentrations.

An analysis of several combinations of input values led to the conclusion that *MRR* is impacted the most by the pressure exerted by the lap plate on the workpiece and by the concentration of the abrasive. This yields the idea to replace the relative speed by the concentration of the abrasive in Preston’s equation for the calculation of *MRR.* Thus, the original formula *MRR = k∙p∙vr* [17] becomes:

(22) $$MRR=K\cdot p^{q}\cdot C^{r}$$

where *K* is an experimentally determined coefficient taking into account the influence of the other input quantities. The exponents *q* and *r* have experimentally determined values and are the mathematical expression of the non-linear dependency of *MRR* on pressure and concentration.

**Figure 10:**
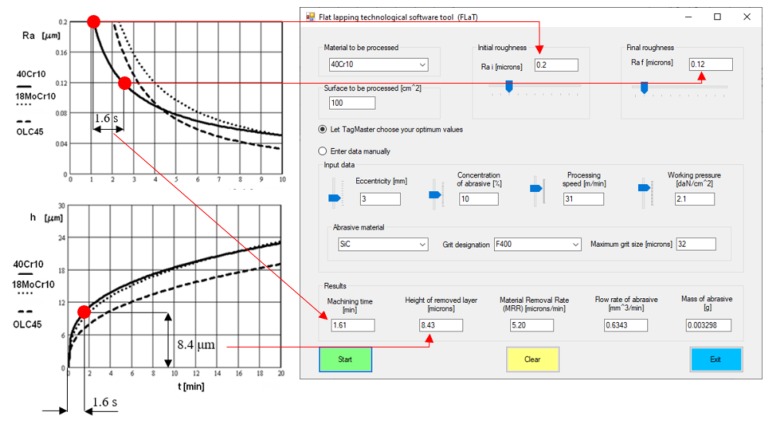
Comparison of the experimental values and those provided by the Flat Lapping Technological Software Tool (FlaT).

The results provided by the Flat Lapping Technological Software Tool (FLaT) were validated based on their comparison to the experimental ones [4]. Thus, for example, Figure 10 shows the graphs obtained upon flat surface lapping of several sheets of steel next to a screen featuring the data provided by FLaT. The coincidence of the experimental values with those provided by the proposed software tool can be noticed.

## 5. Conclusions

Flat surface lapping is a finishing machining process meant to ensure special form and dimensional precision, low roughness, and superficial material layers unaffected thermally. Although known for thousands of years, this method of processing materials does not benefit from thorough theoretical support. To this day, lapping is performed empirically. The quality of the machined surface often differs from one operator to the other, the output quantities depending on the selection based solely on personal experience of the values of the system input parameters. In order to ensure the predictability of lapping results thorough study concerning the interlinking of numerous input quantities is called for, as well as computer-aided working tools.

Within the framework of these requirements, the paper puts forward a number of original contributions useful to industrial specialists. Thus, the paper presents:

- An original methodology for calculating lapping process outcomes, based on the assumption of the spherical model for the abrasive grains. Modeling the flat surface lapping process is also based on the estimation of the percentage of abrasive grains that participate in the actual cutting, presuming a normal distribution of grain sizes.

The spherical model-based working methodology is a quantitative tool that determines the penetration depths of the abrasive grains into the workpiece and the lap plate, the duration of cutting, the necessary machining allowance, the material removal rate, and the abrasive flow rate to be fed into the working area.

- A software application based on the proposed model that provides the engineer with the necessary technical documentation. The application simplifies and significantly reduces the duration for calculating the output quantities representing the optimized machining parameters for flat lapping. This working instrument is validated by the coincidence of the results provided by the proposed software tool with that obtained experimentally.

The utility of this software application consists in the data provided to industrial users, namely output quantities like machining time (min), height of the removed layer of material (μm), material removal rate (μm/min), the flow rate of the abrasive (mm^3^/min), and the required mass of the abrasive (g).

In addition to its practical utility, the software tool for flat lapping (FLaT) also offers swift conclusions as to the influence of the input quantities to the system on the results of machining.
